# Virtual reality stimulation and organizational neuroscience for the assessment of empathy

**DOI:** 10.3389/fpsyg.2022.993162

**Published:** 2022-11-07

**Authors:** Elena Parra Vargas, Aitana García Delgado, Sergio C. Torres, Lucía A. Carrasco-Ribelles, Javier Marín-Morales, Mariano Alcañiz Raya

**Affiliations:** ^1^Institute for Research and Innovation in Bioengineering, Polytechnic University of Valencia, Valencia, Spain; ^2^Fundació Institut Universitari per a la recerca a l'Atenció Primària de Salut Jordi Gol i Gurina (IDIAPJGol), Cornellà de Llobregat, Spain

**Keywords:** organizational neuroscience, empathy, virtual reality, behavioral data, eye-tracking, machine learning, decision-making

## Abstract

This study aimed to evaluate the viability of a new procedure based on machine learning (ML), virtual reality (VR), and implicit measures to discriminate empathy. Specifically, eye-tracking and decision-making patterns were used to classify individuals according to their level in each of the empathy dimensions, while they were immersed in virtual environments that represented social workplace situations. The virtual environments were designed using an evidence-centered design approach. Interaction and gaze patterns were recorded for 82 participants, who were classified as having high or low empathy on each of the following empathy dimensions: perspective-taking, emotional understanding, empathetic stress, and empathetic joy. The dimensions were assessed using the Cognitive and Affective Empathy Test. An ML-based model that combined behavioral outputs and eye-gaze patterns was developed to predict the empathy dimension level of the participants (high or low). The analysis indicated that the different dimensions could be differentiated by eye-gaze patterns and behaviors during immersive VR. The eye-tracking measures contributed more significantly to this differentiation than did the behavioral metrics. In summary, this study illustrates the potential of a novel VR organizational environment coupled with ML to discriminate the empathy dimensions. However, the results should be interpreted with caution, as the small sample does not allow general conclusions to be drawn. Further studies with a larger sample are required to support the results obtained in this study.

## Introduction

Empathy is a multidimensional construct associated with understanding and connecting with the emotional states of other individuals (Christov-Moore et al., 2014). In general, two main dimensions of empathy have been considered: affective and cognitive (e.g., Cuff et al., 2016). Affective empathy supposes a form of emotional experience congruent with what another individual is feeling (e.g., Preckel et al., 2018), while cognitive empathy refers to understanding others’ emotions. The ability to adopt another individual’s perspective (i.e., perspective-taking) has been considered as the hallmark of cognitive empathy (e.g., Kanske et al., 2015).

The operationalization of empathy as a “dual system” helps explain subsequent empathic behaviors toward others’ emotional states (Goleman, 2006; Hoffman, 2008; Shamay-Tsoory et al., 2009; Heyes, 2018; Decety and Holvoet, 2021). For example, empathy grounds prosocial behaviors, such as helping, caring, sharing, and defending another individual or group (Silke et al., 2018). Empathy can also be at the core of many interpersonal processes, such as cooperation, sociability, and social competence (Eisenberg and Miller, 1987), which apply not only to clinical (e.g., Decety, 2020; Vinson and Underman, 2020) or psychosocial levels (e.g., Davis, 2018; Liu and Shange, 2018) but also to organizational levels (e.g., Gentry et al., 2007; Zivkovic, 2022).

In this regard, the current study focuses on empathy processes linked to organizational dynamics (i.e., organizational empathy; Clark et al., 2019). Within organizations, it has been specifically found that empathy is tightly linked to adaptive management in terms of recognizing the emotional states of other organizational members (Rubin et al., 2005; Burch et al., 2016) and facilitating assessments of their interests and motivations (Avolio and Bass, 1995; Somogyi et al., 2013).

Some studies suggest that empathic managers may reinforce employees’ motivation, optimism, and commitment by understanding their needs and emotions (Dubinsky et al., 1995; Barling et al., 1996). Moreover, this empathic management has been suggested to be associated with better outcomes, communication, and decision-making (Rahman and Castelli, 2013).

Therefore, organizational empathy may be understood as the balance between organizational skills, such as decision-making, and empathic abilities (*cf.* Mittal and Sindhu, 2012). From this point of view, being able to estimate high and low empathic profiles accurately can be of great advantage for organizational research, particularly if considering that individuals with similar managerial skills can broadly differ in their empathic abilities (Leiberg and Anders, 2006; Gerdes et al., 2010; Reniers et al., 2011).

The assessments of organizational empathy have mainly relied on self-reports and questionnaires, which present limitations and biases (e.g., Nederhof, 1985; Furnham, 1986; Grimm, 2010). For example, response biases in terms of social desirability or variability among different empathy instruments have been indicated because they do not address the same dimensions of empathy or present poor construct validity (see de Lima and de Lima Osório, 2021). Against this limitation, empathy assessments based on physiological and behavioral data are emerging to complement standard empathy psychometrics (e.g., eye-tracking; Cowan et al., 2014). However, methodological approaches capable of integrating this type of assessment within more realistic organizational settings are still necessary (*cf.* Clark et al., 2019). In this regard, the current study presents an innovative approach to assessing organizational empathy based on a virtual reality organizational environment (VROE) and machine learning (ML) techniques (Alcañiz Raya et al., 2020).

In the next sections, we first overview some issues related to the assessments of organizational empathy. Thereafter, we define our VROE to explore organizational empathy. Finally, we present an exploratory study using ML to estimate organizational empathy from modeling behavioral data in terms of decision-making and attentional data in terms of eye-tracking.

### Organizational empathy assessment

Research on organizational empathy is a relatively novel field focusing on how empathy relates to workplace behaviors and management (e.g., Gerdes et al., 2010; Cropanzano et al., 2017). In this regard, a recent review on organizational empathy by Clark et al. (2019) highlights several key issues related to the measurement of empathy in organizational research, which are detailed below.

First, most studies on organizational empathy rely on a canonical operationalization of empathy that equates empathy with sympathy (*cf.* Hershcovis and Bhatnagar, 2017). For example, the Interpersonal Reactivity Index by Davis (1980) is a widely used instrument within organizational research that follows that rationale. Sympathy can be understood as a form of empathic response (e.g., Gonzalez-Liencres et al., 2013) that relates feelings of alleviating another individual’s suffering (e.g., Davis, 1983). However, sympathy does not necessarily require feeling a congruent emotional state of others as for affective empathy. Moreover, sympathy only addresses suffering, which excludes emotional states with positive valence (*cf.* Bloom, 2016); precisely for this, instruments capturing both positive and negative affectivities may be adequate for measuring the affective empathy dimension (e.g., López-Pérez et al., 2008).

Second, studies evaluating cognitive empathy rely mainly on perspective-taking. However, cognitive empathy aspects, including emotional understanding or interpretation of facial expressions (e.g., Drimalla et al., 2019), which are linked to cognitive empathy, are less commonly investigated within organizational research (e.g., Besel and Yuille, 2010). This gap also leads to the following important point.

Third, most studies focus on empathy at the trait level (i.e., the tendency to be empathic across different situations). However, empathy can also operate at the state level as responsive to situational cues (e.g., Toomey and Rudolph, 2018). Clark et al. (2019) recommend deepening the study of behavioral empathy. In this regard, behavioral and implicit processes can play a significant role. Explicit behaviors occur through conscious executive control as the outcome of the previous relevant information processing, such as when making a decision within organizational contexts (*cf.* Jackson et al., 2005; Becker et al., 2011). Unlike explicit processes, implicit processes are relatively automatic and outside of conscious control and awareness. Implicit measures of empathy have included both brain and physiological measures, such as electroencephalogram (EEG; Balthazard et al., 2012; Alimardani et al., 2020; D’Errico et al., 2020), galvanic skin response (GSR; Nikula, 1991; Marci et al., 2007; Sequeira et al., 2009), and electromyogram (Brower et al., 2015).

Herein, we draw particular attention to eye-tracking and decision-making. First, paying attention to socially relevant cues, such as body postures, and to facial expressions (e.g., Jolliffe and Farrington, 2006; Besel and Yuille, 2010) mainly provides essential information for decoding other people’s emotional states (Cowan et al., 2014; Hedger et al., 2018) and facilitates the understanding of others’ emotional states (Frischen et al., 2007). People with attentional impairment can also show deficits in empathy-related processes (Gu et al., 2013). Therefore, measuring eye-tracking enables the analysis of the in-depth processes of an individual’s visual attention in social situations and complex simulations. Second, decision-making patterns offer valuable information on the level of empathy. According to the decision-making theory of Rowe and Boulgarides (1983), decision-making can be understood as a continuum linked to how an individual understands and perceives a social situation. In line with this reasoning, individuals oriented toward people and the team present a cooperative decision-making style. In contrast, individuals who focus primarily on achieving their own or organizational goals without considering others present a competitive decision-making style (e.g., Mukherjee and Upadhyay, 2018). Both decision-making styles differ according to the emotional understanding of the situation, which leads to different behavioral patterns: Individuals tending to cooperative decision-making would be concerned with maintaining good relationships, offering support and encouragement to team members, promoting collaboration, and achieving consensus. Meanwhile, individuals tending to competitive decision-making could show marked authoritarian behaviors and make unilateral managerial decisions (Weinberger, 2009). According to Scott and Bruce (1995), people can show behaviors of both styles; however, one of these decision-making styles is usually predominant (e.g., Bruine de Bruin et al., 2020).

Incorporating and combining measures of eye-tracking and decision-making with appropriate psychometrics could provide greater accuracy and validity when evaluating empathy in general and particularly in contexts addressing organizational behaviors.

Finally, Clark et al. (2019) reported that ML could be a methodological approach critical for assessing empathy within organizational contexts because it handles a substantial amount of data. To the best of our knowledge, this is an aspect very scarcely investigated within organizational empathy research and virtual reality (VR).

Against this background, the present study considers the above-mentioned recommendations to explore whether and how eye-tracking and decision-making can be modeled to estimate high and low empathic profiles. We approach this question based on a VR framework.

### Virtual reality and behavioral stealth assessment

Virtual reality can be conceptualized as a synthetic three-dimensional environment that simulates real-life experiences where participants can interact with the environment as if they were in the real world (Pratt et al., 1995; Lumsden et al., 2016). Combining different sensory modalities (e.g., visual, auditory, and haptic) with tracking systems that accurately reproduce the stimuli allows a great sense of presence (e.g., Diemer et al., 2015) and engagement. This sense of presence or “being there” causes the user to be less aware of the unreality of the situation and experience it as if it were real life (both mentally and physically). For example, VR can facilitate decision-making responses as if they were natural analogs (*cf.* Burdea and Coiffet, 1994; Slater, 2009).

Nonetheless, VR applied to empathy has focused more on the training and development of empathy than on its evaluation (Rueda and Lara, 2020). Specifically, the term “empathy machine” has been coined to refer to VR potential to improve the emotional understanding and perspective-taking of others (Bujić et al., 2020; Hassan, 2020). For example, virtual environments have been designed to investigate perspective-taking by enabling “being” in the body of another person (Botvinick and Cohen, 1998; Maselli and Slater, 2013). Within organizational research, a similar approach has been adopted to explore perspective-taking from the viewpoint of managers within a work meeting (Chirino-Klevans, 2017).

Recent meta-analyses (Ventura et al., 2020; Martingano et al., 2021) aimed to investigate and clarify existing research on VR as a means of generating empathy. Ventura et al. (2020) revealed significant positive changes in perspective-taking outcomes after exposure to VR. In contrast, Martingano et al. (2021) revealed that VR improved emotional empathy but not cognitive empathy. Therefore, previous results on cognitive and affective empathies elicited in VR simulations are contrasting.

Importantly, VR allows the integration of stealth assessments (Shute et al., 2009, 2016), which refer to the possibility of capturing behavioral data related to specific skills and attributes, providing indirect evaluations in real time (Mislevy et al., 2003; Shute, 2009). This approach specifically aims to measure performance by unobtrusively logging user behaviors (e.g., time to complete tasks, number of attempts to complete the entire experience, and paths taken to solve a problem) rather than by explicitly asking users to self-report their thoughts and behaviors in an evaluative assessment. Accordingly, this approach is suitable for assessing real-time decisions (e.g., timing and type) and eye-tracking within a virtual organizational context. Although this type of evaluation is valid in many contexts and not only in VR, this technology allows it. The need for the transfer to real life to be as large as possible is simultaneously determined in the current study; VR was used, allowing the collection of metrics, including eye-tracking, in a much more ecological manner. This metric is covertly collected by the HTC system used in the environment presentation (Alcañiz et al., 2018; Parra et al., 2021, 2022).

The stealth method relies on evidence-centered design (ECD), a conceptual framework that can be used to develop assessment models (Shute, 2011). According to the ECD, three conceptual models must be developed before the design of a VR experience:

- Competency model: The identification of competencies aims to describe the set of skills and competencies that must be evaluated. It involves identifying the latent components and their relationship with other constructs to be studied. This study mainly focuses on relationships between empathy measured as a trait (psychometrically) and situations (real-time stealth assessments).
- Evidence model: The actual behaviors that can elicit the competencies to be assessed are identified. For each theoretical competency, various behaviors interacting with responses to a specific problem are individualized and described. The present study proposes the investigation of how decision-making behaviors and eye-tracking (e.g., gaze patterns and eye-fixations) within a work context relate to affective and cognitive empathies.
- Task model: Tasks or situations capable of eliciting behaviors related to the competencies that must be evaluated are created. These tasks are addressed in the following sections.

Therefore, the current study explores the capabilities of a VROE based on the ECD to evaluate empathy through the collection of real-time data related to eye-tracking and decision-making. Furthermore, ML is used as a methodological approach to model the data.

### Machine learning

Machine learning is a scientific discipline within artificial intelligence that designs and develops algorithms that allow computers to unravel cognitive and behavioral patterns from large amounts of empirical data (Mikalef et al., 2018). In particular, ML algorithms can identify and estimate data trends and patterns by building on existing information and highlighting unexpected relationships between variables (Vieira et al., 2017; Alcañiz Raya et al., 2020; Graham et al., 2020). This “learning-by-processing” approach has a great potential to produce accurate predictive models. Recently, an increasing number of studies within the organizational field have implemented ML techniques applied to large amounts of data (George et al., 2014; Leavitt et al., 2021). For example, ML has been used for the assessment of candidates (Faliagka et al., 2012) and identification of leadership roles (Doornenbal et al., 2021) as well as personality traits of managers (Hrazdil et al., 2020). Machine learning has also been used to study communication skills in the workplace (Suen et al., 2020) and evaluate gaze patterns and facial expressions (Muralidhar et al., 2018).

As previously introduced, there is an increasing urge to advance this type of methodology within organizational empathy research (e.g., Clark et al., 2019). Our study thereby presents a novel VR framework to investigate this issue.

### New integrated approach to organizational empathy assessment

This study explores the feasibility of a VROE to investigate organizational empathy from both explicit and implicit data. At the theoretical level, the study aimed to link trait and situational empathy assessments by using a psychometric instrument covering different dimensions of empathy (cognitive and affective). Decision-making and eye-tracking data measured in real-time within the virtual environment are used to assess situational empathy. At the methodological level, an ML approach is implemented to model the data.

Accordingly, the study raises the following two research questions:

RQ1: How can decision-making and eye-tracking data be integrated within an organizational virtual environment to assess situational empathy?
RQ2: Can ML techniques discriminate high or low empathy from decision-making and eye-tracking data?

## Materials and methods

### Participants

The study sample consisted of 82 Spanish participants (men: 67%, women: 33%; mean = 42, standard deviation = 3.44). The inclusion criteria were the absence of mental disorders or psychiatric medication. All participants signed a written informed consent prior to participation in the study.

### Trait empathy assessment

The Cognitive and Affective Empathy Test (TECA; López-Pérez et al., 2008) was used to measure trait empathy. It consists of 33 items rated on a 5-point Likert scale (1 = “I totally disagree” to 5 = “I totally agree”) representing four subscales. Two of these subscales evaluate cognitive empathy: perspective-taking (eight items; e.g., “I try to understand my friends by looking at situations from their perspective”) and emotional understanding (nine items; e.g., “I notice when someone tries to hide their true feelings”). The other two subscales evaluate affective empathy, both positive and negative: empathetic joy (eight items; e.g., “When something good happens to someone, I feel happy”) and empathetic stress (eight items; e.g., “I cannot help but cry with the testimonials of unknown people”). The Cronbach’s alpha values were 0.70, 0.74, 0.78, 0.75, for the TECA perspective-taking (TECA PT), emotional understanding (TECA EU), empathetic stress (TECA ES), and empathetic joy (TECA EJ) subscales, respectively. Table 1 shows a detailed description of the subscales.

**Table 1:** Description of the dimensions of the TECA questionnaire.

**Scale**	**Subscale**	**Definition**	**High level**	**Low level**
Cognitive dimension	Perspective-taking	Intellectual or imaginative ability to put oneself in the place of another person	They have ease of communication, tolerance, and interpersonal relationships. They also have flexible thinking in such a way that they can adapt their way of thinking to different situations.	They tend to have less flexible thinking and may be less able to understand the mental states of others, which can be a certain obstacle in communication and relationships with other people.
	Emotional understanding	Ability to recognize and understand the emotional states, intentions, and impressions of others	They have great facility for emotional reading in the face of the verbal and non-verbal behaviors of others. At the intrapersonal level, they tend to show greater emotional regulation.	They have difficulty recognizing and understanding the emotional states of others. They also have poorer social skills.
Affective dimension	Empathetic joy	Ability to share the positive emotions of another person	They easily rejoice in the successes or positive events that happen to others, which is related to a good quality of social network.	They have a less tendency to share the positive emotions of others. Being emotionally out of tune is related to having access to a low-quality social network.
	Empathetic stress	Ability to share the negative emotions of another person	They tend to have quality social networks and be emotional and warm in their interpersonal relationships, perhaps with a certain tendency to become overly involved in the problems of others.	They are not easily moved, unemotional, and emotionally distant.

The instrument defines and interprets high and low empathy ratings.

### Situational empathy assessment

Situational empathy was assessed based on decision-making behaviors and attentional patterns *via* eye-tracking features. A series of features regarding these variables are described in Tables 2, 3, respectively.

**Table 2:** Decision-making variables.

Type of variables	Number of features
Participant’s answers during the meeting points (3 × situations 1 and 2 + 2 × situations 3 and 4 + mode)	11
Problem-solving individually (3 × situations 1 and 2 + 2 × situations 3 and 4)	10
Emotion recognition (1 × situations 1–3)	3
Location the participant selects on the meeting table (1 × situation + mode)	5
Messaging application:	
Usefulness score (13)	16
Number of times opened	
Number of messages sent	
Real interaction (times opened – messages sent)	
Mini-game (6×):	18
Number of times selected	
Self-rating of the performance	
Reported reason for selecting the mini-game	

**Table 3:** Description of the eye-tracking variables.

Type of variable	Number of features	Description
Eye fixation	10	Mean number (and standard deviation) of fixations conducted per situation and total value during the entire experience
Inclusive gaze (per situation)	20	Per situation (4×), the average time (s) the participant looked at each virtual agent (6×) while speaking
Inclusive gaze (average)	5	Over the entire experience, the average time (s) spent by the participant talking while looking at each virtual agent (6×)
Averted gaze (per situation)	60	Per situation (4×), the average time (s) the participant looked at virtual agent B while virtual agent A was speaking (6×)
Averted gaze (average)	15	Over the entire experience, the average time (s) the participant looked at virtual agent B while virtual agent A was speaking

The environment is considered as another possible area to look at while speaking; thus, the time spent looking at the environment is also calculated, as it is a virtual agent itself.

### Experimental procedure

The participants completed the TECA online with a short demographic questionnaire before the experimental testing at the laboratory. The experimental testing consisted of a 1.5-h session in which the participants experienced a workplace dynamic in an immersive VROE. The participants were seated and wore a head-mounted display (HMD) equipped with an eye-tracking feature, calibrated at the beginning of the session. Thereafter, the VROE experience began. The first 2 min showed a brief tutorial explaining how to use the virtual environment. The participants then went through activities taking place either at an office or a meeting room.

Visual attention was measured using the HTC VIVE Pro Eye HMD, with a combined resolution of 2,880 × 1,600 pixels (1,440 × 1,600 per eye), a field of view of 110°, and a refresh rate of 90 Hz. The VROE was developed using the Unity 5.5 software, applying C# programming language with the Visual Studio tool. The VR application ran on the MSI GE75 Raider 9SF-1204XES laptop (17.3″, i7-9750H, RAM 32 GB, 1 TB NVMe PCIe Gen3x4 SSD, GeForce RTX 2070 GDDR6 8GB).

### Virtual reality organizational environment description

**Figure 1:**
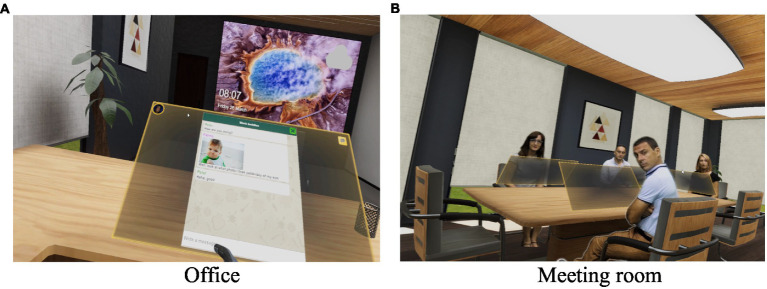
Scenarios of the virtual reality environment.

Virtual scenarios and decision-making tasks potentially related to empathy and suitable workplace settings were designed following the ECD guidelines. The virtual environment consisted of four situations with the same organization each. Specifically, the design focused on two main scenarios: (1) an office and (2) a meeting room (Figure 1). The main difference between both scenarios was their social character. In the office, the participants were alone, performing a series of tasks individually. In the meeting room, they shared a table with four other co-workers. These co-workers were actors pre-recorded using the Chroma key technique. This technique allows cutting the part of a video that is intended to be integrated into the virtual environment; in this case, different actors were recorded saying the designed dialog.

#### Tutorial

At the beginning of the experience, a tutorial was provided. This tutorial showed relevant aspects to the participant to move around the environment and to interact or respond to the different types of interfaces proposed throughout the experience. The user was expected to become familiar with the virtual environment during this learning period, with no metrics collected.

#### Virtual experience at the office

The participants started the virtual experience within the office. They adopted the role of a new worker within the organization. After a brief period to familiarize themselves with the environment, the participants were kindly asked (by a pop-up message) to sit at a chair in front of a table with a computer inside the virtual office. This message was presented directly in the virtual environment. The entire experience was within the virtual environment during all situations. Herein, it was explained that the computer could involve two interactive tasks: chatting with co-workers and answering email messages.

- Chatting with co-workers: The goal of this activity was to evaluate decisions framed within a chat group with other colleagues. Concretely, the participants were to decide freely whether to chat. The users interacted with the chat through a virtual keyboard. Examples of chat topics were internet jokes and comments toward images with humorous content or comments on others’ personal problems. Decision-making in terms of the number of times that the participants opened the chat, answered, and sent messages was evaluated.
- Answering emails: Some emails were framed in a narrative attempting to capture the empathic skills of the participants. To be more precise, the main objective of this activity was recognizing and understanding emotions by paying attention to verbal and non-verbal cues. For example, an email addressed a recruitment task where candidates’ images were blurred so that their faces were not clearly visible. The participants were to answer questions referring to emotion recognition. Another email sent a video showing a man talking and gesturing. However, the narrative addressed problems with the audio. Accordingly, the participants’ attention toward body language was tracked as a potential indicator of emotional understanding.

#### Virtual experience at the meeting room

**Figure 2:**
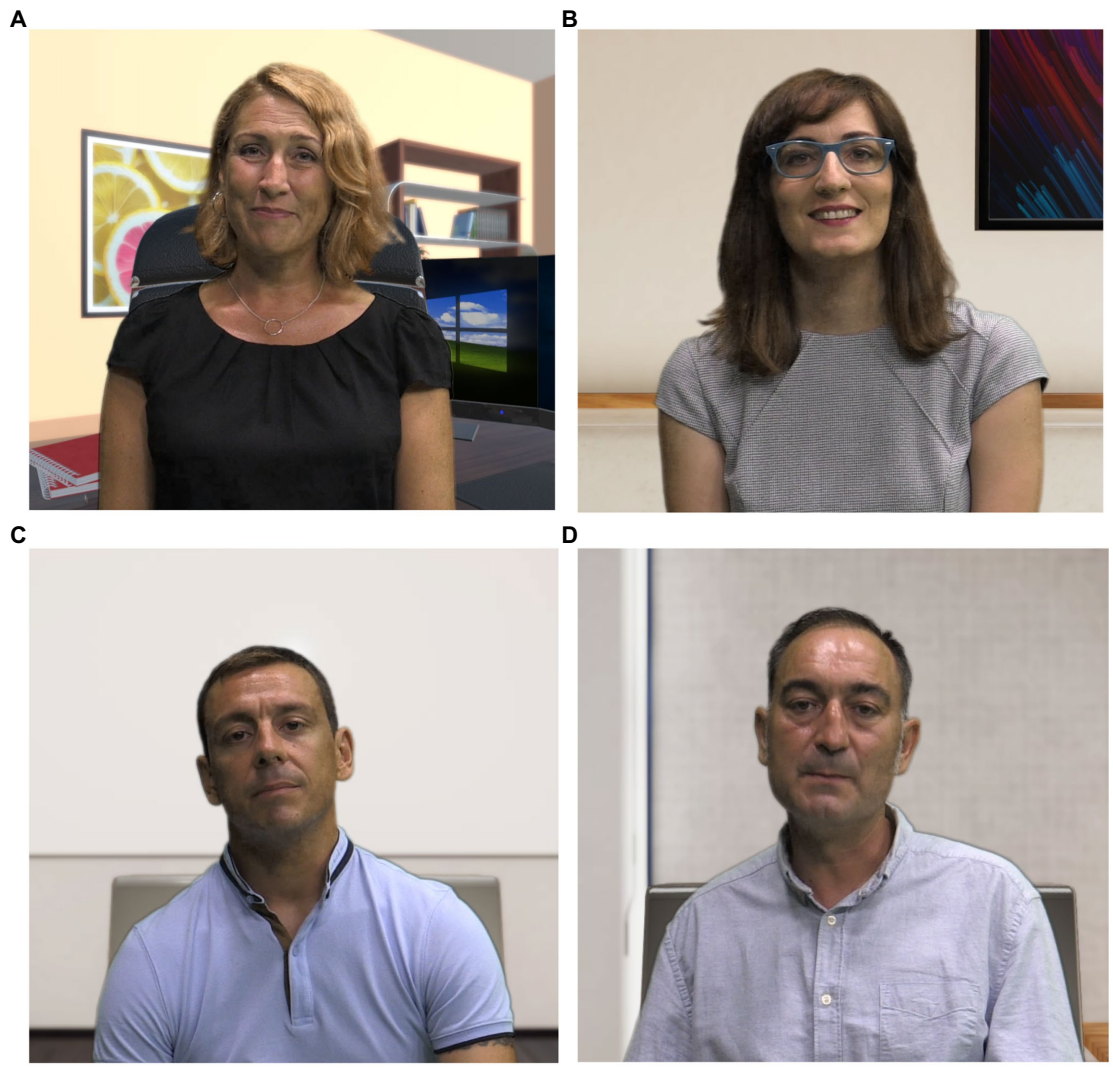
Virtual agent characters: **(A)** organizer agent, **(B)** communicative agent, **(C)** passive agent, and **(D)** logical agent.

Once the above-described tasks at the office were finished, the participants entered the group meeting scenario. This scenario involved four virtual agents (two women and two men) with different attitudes and behavioral profiles. Specifically, one of the characters was presented as the organizer, another as communicative, another as logical, and the remaining as passive (Figure 2):

- Organizer agent representing the role of the primary manager: A woman showed planned, sequential, and structured thinking. Her role focused on deciding what steps to take after a problem-solving debate.
- Communicative agent representing the role of the human resource manager: A woman showed interpersonal warmth, fluid communication, and holistic thinking. This agent showed self-confidence and a predisposition to understand others’ points of view. Moreover, she encouraged everyone to yield consensus regarding the discussed topic. This character was sensitive to both personal and organizational problems.
- Passive agent representing the role of the production department head: A man showed a non-interventional attitude. He avoided providing any feedback regarding the discussed topic and left the decision on the discretion of the other members. However, he exalted when the topic referred to his department.
- Logical agent representing the role of the sales department head: A man showed technical and analytical reasoning and a pessimist tendency. He did not show empathetic attitudes toward the rest of the characters. On the contrary, he showed a distant, critical, and competitive attitude. Additionally, this agent set clear standards to follow and punished any mistakes.

At the beginning of the meeting room scenario, the virtual agents were sitting at a table and talking. The organizer agent invited the participants to join them at the table. Herein, the participants were to decide where to sit among three free chairs and move in the scenario through a teleportation paradigm. A problem was then presented by one of the virtual agents. The participants listened first to the opinions of the other members and were then asked to explain their opinion about the problem. They were prompted to talk and explain their arguments by voice to be more realistic and engaging and choose a solution to the problem by clicking on a list of four alternatives. Finally, the organizer noted the participants’ decision and closed the debate. The meeting topics had different emotional connotations. For example, one topic addressed the organization of a meeting, whereas another meeting addressed a heated discussion concerning issues associated with the role of the participants.

The participants’ decisions addressed the following different behavioral styles:

Decision style 1: Cooperation was sought, reaching agreements among all members. Interest in the welfare of others was appreciated, and emotional responses were provided to demands. The following is an example: “We could focus on deciding who will be responsible. What do you think? Do you think we can distribute the tasks as I propose?”
Decision style 2: This style involved not making decisions unless the opinion of others was known, which was explained by an excessive concern for rejection. Decisions showed high sensitivity to the emotions of others. However, extreme rejection concerns may leave few cognitive resources to understand such emotions. The following is an example: “Perhaps I have been here too little time to be able to divide the tasks. I think it would be advisable for you to make the decision this time.”
Decision style 3: This style was characterized by rapid and rigid responses. Decisions reflected minimal trust in others and dislike when the rest of the team disagreed. Decisions did not reflect a willingness to understand or respond appropriately to the emotions of others. However, there were a high fear of rejection and a need for approval. The following is an example: “From my point of view, the best distribution is this.”
Decision style 4: This style was defined by a complete lack of interest in others and a lack of cooperation and support. No interest or concern for others was reflected, and willingness to take another person’s perspective nor the ability to share another person’s emotions was not appreciated. The users distanced themselves abruptly or stopped collaborating because they felt pressured. The following is an example: “Maybe we should go to the next point of the meeting and decide later.”

During the different meetings, chat messages (as previously described) were also implemented. At the end of the meetings, a series of mini-games were implemented as filler tasks. Depending on the decision, there were games of logic, creativity, and cognitive load.

After decision-making about each meeting, another decision had to be made individually regarding other situations. The purpose of these tasks was to collect decision-making styles in situations that may have emotional and labor repercussions on the rest of the co-workers without counting on their opinion. The following is an example: “You have pending tasks, but your workload is very intense this week, and you have decided to put some of your work aside. How would you do it?” The participants had to select from four options regarding the decision that best fit their decision style (e.g., “You tell the employees to do the job and leave them free to decide how to do it” vs. “You select two different employees to each do the task, and finally, you select the best job”).

#### Virtual reality at the office

After resolving the last point of the meeting, the participants returned to the office, where they were asked to rate their behavior and involvement in the group chat and their performance in the problem-solving tasks. In addition, the participants were shown a variety of mini-games. They were encouraged to select the games they wanted to play (e.g., logic, creativity, or mental speed). Once they had played, the participants had to indicate the reasons for the selected option, rate their performance level, and evaluate the mini-games as positive or negative.

In brief, a virtual environment that aimed to stimulate behaviors linked to different levels of empathy was designed using avatars with different personality traits and personal and work decision-making tasks.

**Figure 3:**
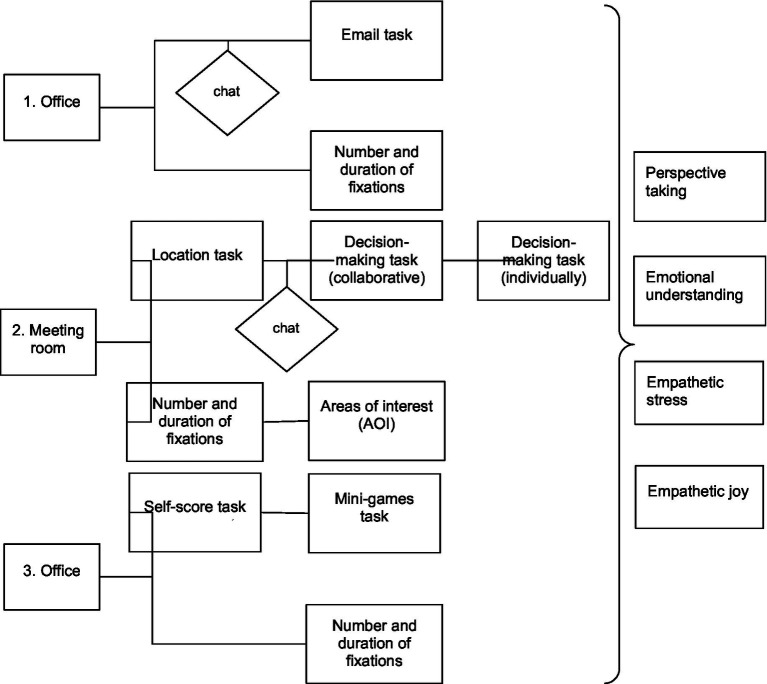
Structure of the virtual environment.

Figure 3 displays the structure of the virtual environment as well as the information collected from both sources (decision-making and eye-tracking).

### Data processing

Data were obtained from three different sources: the participants’ answers to the TECA questionnaire, behavioral data (i.e., decisions made by the participants during the VR experience), and eye-tracking data (i.e., gaze fixations). Raw data were used to obtain a set of variables. Specifically, a total of 63 variables represented the decision-making data (Table 2), whereas a total of 110 variables represented the eye-tracking data (Table 3).

### Statistical analysis

Statistical analyses were performed using R (version 3.6.1). Eight participants did not answer the TECA questionnaire; thus, their data were excluded from the analysis. A multivariate outlier analysis (Filzmoser, 2004) considering the four dimensions of the questionnaire was performed. In this outlier detection method, the distance between the participants was calculated by considering all subscales of the questionnaire and estimating the probability of this distance belonging to a chi-square distribution. When the probability was below 0.01, the participants’ scores were defined as outliers. Accordingly, two participants were excluded from further analysis. Finally, 72 participants were considered.

Mean, median, minimum, maximum, and standard deviation values and interquartile ranges were used to describe the TECA scores. The normality of the scores was inspected using the Shapiro–Wilk test. Statistical significance was defined at *p* < 0.05.

### Machine learning

Machine learning was used to explore the potential to discriminate between high and low empathic profiles from the decision-making and eye-tracking assessments during the VR experience. Accordingly, the TECA empathy scores were categorized into high or low according to the median value in each subscale (i.e., TECA PT, EU, EJ, and ES). The ML-based models were then trained per each subscale to select the best set of features characterizing each of them.

Initially, feature selection using a backward sequential wrapper (Doak, 1992) was performed to reduce the number of features. The method started by building a model based on a particular ML algorithm with all available features and measuring its performance. Thereafter, a feature was removed at each step; the model was re-trained; and its performance was measured. The feature whose removal increased the performance measure the most (i.e., Cohen’s Kappa) was removed from the set of features used in the next step. After several steps in which the performance metric did not vary by more than 0.01, the process stopped.

Different ML algorithms were used to obtain the best set of features: random forest, SVM, Naïve Bays, XGBoost (gradient boosting tree), and K-nearest neighbor (kNN). These algorithms used the default hyperparameters defined in the mlr package version 2.14.0 (Bischl et al., 2016). After obtaining the best set of features for each ML algorithm, we trained the model. The accuracy (Cohen’s Kappa), sensitivity (true positive rate), and specificity (true negative rate) of the model were calculated.

Both steps used repeated cross-validation (five folds, four times); thus, the validation metrics corresponded to the mean value across the 20 repetitions. The same folds were used to validate all algorithms. The information from 10 randomly selected participants was excluded from this training model process and was used only as a test set.

## Results

### TECA scores

Table 4 shows the scores in the TECA subscales. The participants scored (mean ± standard deviation) 34.17 ± 4.31 in the TECA EU and 34.89 ± 3.28 in the TECA EJ (Shapiro–Wilk test, *p* > 0.05). The median ± interquartile range of the scores in the TECA ES and TECA PT was 21.5 ± 8 and 32 ± 6, respectively (Shapiro–Wilk test, *p* < 0.05). Once categorized, 55.55%, 63.89%, 50%, and 52.78% of the participants scored high in the TECA EU, TECA EJ, TECA ES, and TECA PT, respectively.

**Table 4:** Scores in each TECA subscale.

Subscale	Mean (standard deviation)	Median	Standard deviation	Interquartile range	Minimum	Maximum	Shapiro–Wilk normality test, *p*-value	High score (*n*)	Low score (*n*)
TECA EU	34.17	35	4.31	6.25	23	42	0.009	40	32
TECA EJ	34.89	35	3.28	4.25	26	40	0.006	46	26
TECA ES	21.81	21.5	5.62	8	10	39	0.37	36	36
TECA PT	31.90	32	3.85	6	22	40	0.46	38	34

EU, emotional understanding; EJ, empathetic joy; ES, empathetic stress; PT, perspective-taking. The last two columns show the number of participants in each category after discretizing the scores according to the median score in each subscale.

### TECA recognition models

The metrics of the best ML-based models for each TECA subscale and their characteristics are shown in Table 5. The best model for the TECA EU was based on the kNN, which selected 19 features (63.16% from the eye-tracking data and 36.84% from the behavioral data) and achieved an accuracy of 69% in the validation set and 58% in the test set. The model for the TECA EJ was built using random forest, which selected 14 features (28.57% from the eye-tracking data and 71.43% from the behavioral data) and achieved a similar accuracy between the validation and test sets (76% and 75%, respectively). The model for the TECA ES was built using random forest, which selected 17 features (94.11% from the eye-tracking data and 5.88% from the behavioral data) and achieved an accuracy of 81% in the validation set and 67% in the test set. The model for the TECA PT was built using random forest, which selected 19 features (73.68% from the eye-tracking data and 26.32% from the behavioral data) and achieved a similar accuracy between the validation and test sets (82% and 83%, respectively).

**Table 5:** Metrics of the best model achieved for each TECA subscale both in the validation and test sets.

Subscale	Model	Features (*n*)			Validation set					Test set				
		Eye-tracking	Behavioral	Total	Accuracy	Kappa	AUC	TPR	TNR	Accuracy	Kappa	AUC	TPR	TNR
TECA EU	kNN	12	7	19	0.69	0.3	0.67	0.81	0.51	0.58	0	0.44	0.75	0.25
TECA EJ	Random forest	4	10	14	0.76	0.49	0.81	0.87	0.65	0.75	0.47	0.83	0.86	0.6
TECA ES	Random forest	16	1	17	0.81	0.58	0.85	0.88	0.71	0.67	0.31	0.66	0.71	0.6
TECA PT	Random forest	14	5	19	0.82	0.63	0.87	0.83	0.83	0.83	0.67	0.83	0.83	0.83

The number of variables used by each model is divided according to the source (i.e., eye-tracking or behavioral data). The values shown per metric in the validation set are the mean values of the cross-validation iterations. TPR, true positive rate; TNR, true negative rate.

## Discussion

This study explored the links between trait and situational organizational empathies through a novel VROE. Behavioral (i.e., decision-making) and attentional data (eye-tracking) were measured in real time during the VR experience and subsequently modeled through ML techniques to estimate the cognitive and affective dimensions of trait empathy. Concretely, the TECA questionnaire was used to assess cognitive empathy encompassing perspective-taking and emotional understanding and affective empathy encompassing empathic stress and empathetic joy.

The VR experience was designed following the ECD, which enabled the collection of behavioral decision-making and eye-tracking data associated with each empathy dimension. Machine learning was used to build different models based on these two sources of information recorded during the VR experience, making it possible to identify the behavioral decision-making and eye-tracking variables that best define and predict each of the dimensions and to perform an analysis of the frequency distribution (high vs. low) of the different empathy dimensions.

This multi-method approach combining VR and behavioral and attentional measures offers a deeper understanding of the psychological construct to be evaluated and its manifestations. In addition, it improves the ecological validity of the use of self-report measures alone, since it enables behavioral decision-making data to be captured in scenarios that simulate real management situations.

### High and low perspective-taking, emotional understanding, empathetic stress, and empathetic joy

The first objective was to identify the differences between the different empathy dimensions both in the traditional measure questionnaire and in the VR experience. The analysis of the traditional measures indicated that for the self-report form, 56% of the participants had a high score for emotional understanding; 64%, empathetic joy; 50%, empathetic stress; and 53%, perspective-taking. These results indicate that the traditional evaluation measures can define and classify each of the empathy dimensions. Furthermore, the number of participants with high scores was higher than that of those with low scores for emotional understanding and empathetic joy; the number of participants who scored high and low for empathetic stress was similar; and the number of participants who scored low was higher than that of those who scored high for perspective-taking.

Regarding the VR experience, the results supported both research questions: The different dimensions of empathy could be differentiated by the gaze patterns and behaviors elicited during the immersive VR experience. However, most models were based mainly on the eye-tracking data rather than on the behavioral data, except for the empathetic joy dimension. In fact, with this dimension excluded, the eye-tracking data were represented between 63% and 94% for all models. Meanwhile, the behavioral data were represented between 6% and 71% in the selected variables for all models.

Therefore, eye-tracking played a more relevant and distinctive role in predicting the different empathy dimensions than did decision-making during the VR immersion. This could be explained by the idea that it is necessary to accurately attend to the surrounding context to start empathic processes (Frischen et al., 2007). This information is acquired by observing and paying attention to behaviors and facial expressions, which allow the detection of complex mental states, such as intentions, thoughts, beliefs, emotions, and desires of those around (Balconi and Canavesio, 2016). Therefore, through gaze, individuals attempt to accurately assess the motivations, intentions, and emotions to anticipate the behavior of another and to amend own decisions and actions accordingly (Singer, 2006).

Nevertheless, the decision-making variables also added value to the ML-based models, supporting the idea that empathy is a precursor to prosocial behavior, including any action performed to alleviate and share positive and negative emotions, which have a repercussion in all fields of work. Especially in management environments in which decisions have to be made under uncertainty, risk, and stress, empathy is especially important, since it favors the maintenance of social relationships and encourages people to serve the needs of others (Vyatkin et al., 2019).

Based on the ML-based model metrics, the feasibility of the virtual environment to track behaviors (eye-gaze patterns and behavioral decision-making) enables the classification of participants according to their level of empathy dimensions. However, our results suggest that empathy can be better identified from eye-tracking than from decision-making. Eye-tracking-related variables were systematically selected more frequently for all empathy subscales, except for the TECA EJ, which presented more behavioral variables. This could be attributed to the fact that people could be more extrinsically motivated to respond behaviorally to positive affects than to negative affects. According to Telle and Pfister (2016), empathy from positive events implies low costs but high benefits: The experience of a pleasant emotional state is acquired. While empathy from negative events generates unpleasant feelings, activating prosocial behaviors can yield costs for the individual. The TECA PT and EJ models were the best models both in terms of absolute accuracy and similarity between the results in the validation and test sets. The TECA ES model also showed similar results in the validation set but poor results in the test set. Finally, the TECA EU model was unable to generalize the results in the test set.

### Theoretical and practical implications

The main added value of this study lies on the detection and assessment of empathy by integrating (a) behavioral information (eye-tracking and decision-making); (b) a highly ecological and standardized setting, such as VR; and (c) a powerful method capable of analyzing an extensive amount of data and predicting models—ML. Therefore, this study yielded better understanding of the practical implications and benefits of using implicit measures in general (e.g., EEG, GSR, and heart rate variability) and eye-tracking and decision-making data in particular. Based on the findings, both implicit measures allowed the measurement of empathy in a more ecological manner, offering valid data during the VR experience. This study also confirmed the relevance of ML in identifying and predicting data trends and patterns from information on VR performance.

This multi-method approach can increase the knowledge about the attentional and behavioral patterns and decision-making processes conducted by workers with different empathy levels in complex work situations. In addition, unlike most evaluations that use subjective self-report measures, this method combines neuroscience with VR, which attributes greater objectivity and ecological validity to the results. Regarding implicit measures, the importance of non-verbal cues in identifying empathy characteristics, especially ET measures that cannot be evaluated through explicit measures, is highlighted.

This study also advances research on the evaluation of empathy in VR environments, since most studies focus on improving and training this competence rather than on its evaluation (e.g., Bertrand et al., 2018; Dyer et al., 2018). This explains why most studies focusing on empathy assessment through implicit measures use visual stimuli, such as images or videos, to evaluate gaze patterns instead of experiential stimuli (e.g., Zhi-Jiang and Pan-Cha, 2016; Nebi et al., 2022). The present study contributes to the use of experiential stimuli by offering a novel multi-method approach that assesses empathy, electing its manifestation through exposure to VR situations strategically designed for this purpose and recollecting data from eye-tracking and decision-making patterns.

The current study also provides a broad overview of the benefits of using ML as a dataset analysis methodology. This methodology allows the identification of hidden patterns between different apparently unrelated variables, which may be of interest for the hidden evaluation of empathy in a virtual environment whose apparent objective is not the evaluation of such. Thus, it can be constituted as an alternative to other methods of assessing empathy, which may serve as an initial tool for the assessment of empathy in work environments prior to the training phase in the future. After the training phase, this assessment tool could be used again to check the efficacy of the training. The methodology could be applied for the assessment of other physiological constructs in clinical and organizational areas.

## Limitations and future directions

In this study, we identified some limitations that could be helpful for future research on empathy and the organizational field. First, our ability to generalize the results was restricted by the small number of the participants included (*n* = 71). Thus, the size of the test set for the ML-based models was also small. Second, we built the high and low target variables based on the mean or median values of the responses from the study; thus, they may not be extrapolated to the rest of the population. This indicates that both conditions could compromise the generalizability of the theory. However, the main objective of the study was not to design an evaluation tool that would replace traditional selection tools, such as questionnaires or interviews, but to explore the feasibility of designing an empathy evaluation strategy more ecologically, replicating the results of the TECA by capturing behavioral measures. This goal was achieved by using ML, which allowed the creation of a predictive classification model. To our knowledge, our study is the first to integrate VR, implicit measures, and ML to explore and assess empathy dimensions in a specific population.

Regarding future directions, this work can serve as a basis for the study of psychological constructs, including empathy, using a novel technology (e.g., VR with implicit measures and ML) to make predictions. Increasing the number of participants in future research and including an expert judgment on the levels of empathy presented by candidates to improve data validity are recommended.

## Conclusion

Based on the current results, we conclude that behavioral measures captured during VR experiences constitute valid parameters for detecting and assessing different empathy dimension levels. ET measures provide the core information in the classification models. Therefore, this multi-method approach consisting of an immersive VR system, eye-tracking, and ML offers a novel perspective on the study of empathy and the ability to replicate the results of the TECA questionnaire.

## Data availability statement

The raw data supporting the conclusions of this article will be made available by the authors, without undue reservation.

## Ethics statement

This study was reviewed and approved by the Ethics Committee of the Polytechnic University of Valencia (protocol code: P01_08_07_20) and conducted according to the guidelines of the Declaration of Helsinki (1964). The participants provided written informed consent for participation in this study and for publication of any identifiable images or data included in this article.

## Author contributions

EP and MA: conceptualization, methodology, and resources. EP: validation and investigation. LC-R and JM-M: formal analysis and data curation. EP, AG, ST, and LC-R: writing—original draft preparation. AG, ST, LC-R, EP, and JM-M: writing—review and editing. MA: supervision. All authors contributed to the article and approved the submitted version.

## Funding

This work was supported by the European Commission (project RHUMBO: H2020-MSCA-ITN-2018-813234) and by the Generalitat Valenciana funded project “REBRAND” (grant number: PROMETEU/2019/105).

## Conflict of interest

The authors declare that the research was conducted in the absence of any commercial or financial relationships that could be construed as a potential conflict of interest.

## Publisher’s note

All claims expressed in this article are solely those of the authors and do not necessarily represent those of their affiliated organizations, or those of the publisher, the editors and the reviewers. Any product that may be evaluated in this article, or claim that may be made by its manufacturer, is not guaranteed or endorsed by the publisher.
